# The correlation between exercise types and adolescents’ executive function and mobile phone dependence: a cross-sectional study from the perspective of motor skill classification

**DOI:** 10.3389/fpsyg.2026.1695406

**Published:** 2026-02-06

**Authors:** Yingxing Zhang, Rui Hua, Mengyuan Deng, Peng Shi

**Affiliations:** 1 School of Physical Education, Sichuan University of Science and Engineering, Zigong, China; 2 School of Mechanical Engineering, Sichuan University of Science and Engineering, Zigong, China; 3 School of Physical Education, Shandong University of Technology, Zibo, China

**Keywords:** adolescents, executive function, exercise types, mobile phone dependence, motor skills

## Abstract

**Objective:**

This study aims to explore the correlation between different exercise types and adolescents’ executive function as well as mobile phone dependence, and to investigate the mediating role of executive function in the relationship between exercise types and mobile phone dependence.

**Methods:**

This study adopted a cross-sectional study design, and a total of 1,016 first-year and second-year high school students from three cities in Sichuan Province were selected via convenience sampling. The Adolescent Executive Function Scale was used to assess ecological executive function. The Self-Rated Questionnaire for Adolescent Mobile Phone Use Dependence was employed to evaluate mobile phone dependence. Participants were divided into the open-skill exercise group and closed-skill exercise group through questionnaire surveys. Statistical analyses, including generalized linear models, partial correlation analysis, and mediating effect analysis, were conducted using SPSS 21.0 software.

**Results:**

Compared with the closed-skill exercise group, adolescents in the open-skill exercise group had significantly higher inhibitory control (β = 0.410, 95% CI = 0.083∼ 0.738, *P* = 0.014) and cognitive flexibility (β = 0.588, 95% CI = 0.188∼0.988, *P* = 0.004), as well as significantly lower physical and mental impacts caused by mobile phone dependence (β = −0.600, 95% CI = −1.073∼-0.127, *P* = 0.013). In addition, inhibitory control and cognitive flexibility played a partial mediating role in the relationship between open-skill exercises and the physical and mental impacts of mobile phone dependence, with indirect effects of −0.178 and −0.278, respectively.

**Conclusion:**

Open-skill exercises are associated with higher levels of inhibitory control and cognitive flexibility in adolescents, as well as lower physical and mental impacts caused by mobile phone dependence. Moreover, inhibitory control and cognitive flexibility play a mediating role in the association between open-skill exercises and mobile phone dependence. This study provides scientific support for adolescents’ exercise type selection and the formulation of relevant health policies.

## Introduction

Executive function is a core cognitive concept in the fields of psychology and neuroscience. It refers to the advanced psychological ability of individuals to consciously regulate and coordinate their own cognitive processes when completing complex cognitive tasks (Baggetta and Alexander, 2016). It is not a single cognitive skill, but a collection of a series of interrelated cognitive processes, mainly responsible for guiding individuals’ goal-oriented behaviors and helping people solve problems flexibly and effectively in a dynamically changing environment (Cañas et al., 2003; Drigas and Karyotaki, 2019). Different scholars have different definitions of the components of executive function. At present, it is widely recognized that it includes three sub-components: inhibitory control, working memory, and cognitive flexibility (Miyake et al., 2000; Pennington and Ozonoff, 1996). Adolescence is a critical period for the development of an individual’s executive function (Poon, 2018). During this stage, the development of executive functions is of great significance for their academic performance (Ramos-Galarza et al., 2020), mental health (Ajilchi and Nejati, 2017), social adaptation (Holley et al., 2017; Nigg, 2017), and future development (Jurado and Rosselli, 2007). Therefore, paying attention to the cultivation of adolescents’ executive function is of great significance to both individual growth and social development.

Currently, numerous studies (Dong, 2018; Song et al., 2022; Xie, 2020; Yang, 2021) have confirmed that physical exercise can promote the development of executive function in adolescents. These studies (Dong, 2018; Song et al., 2022; Xie, 2020; Yang, 2021) have found that exercise lasting for more than 10 weeks, at moderate intensity, more than 3 times a week, and with single session duration of more than 30 min is more conducive to promoting the development of executive function. In addition, the relationship between exercise types and executive function has also attracted widespread attention from researchers. Motor skills are divided into open skills and closed skills according to the predictability of environmental changes in motor tasks (Zhang, 2012). Among them, open skills refer to the skills of performing motor tasks in an unpredictable environment, where individuals need to respond and adjust their actions according to changes in the environment, such as football, basketball, table tennis, tennis, taekwondo, etc. Closed skills refer to the skills of performing motor tasks in a stable and predictable environment, where individuals can pre-plan their movement procedures, such as tai chi, rhythmic gymnastics, track and field events, etc., (Zhang, 2012). Many studies (Feng et al., 2023; Shi et al., 2022) have found that open skill exercise is more effective than closed skill exercise in promoting adolescents’ executive function.

However, when looking at existing studies, a prominent limitation is that the assessment of executive function mostly relies on standardized tasks in laboratory environments, such as the Flanker task, 2-back task, and More-odd Shifting task. While these assessment methods can accurately measure specific components of executive function, they struggle to truly reflect an individual’s ability to apply executive function in solving practical problems within daily life contexts. In other words, computerized test tasks lack effective evaluation of ecological executive function (Gioia et al., 2002). Ecological executive function emphasizes an individual’s ability to mobilize cognitive resources in combination with specific situations to achieve goals in real, complex natural environments, and this ability is more critical for adolescents’ daily learning and social adaptation. In view of this, it is necessary to more comprehensively and in-depth explore the correlation between exercise types and executive function, with a particular focus on the level of ecological executive function, so as to better promote the physical and mental health development of adolescents.

In addition, with the rapid development of science and technology and the popularization of smartphones, the problem of adolescent mobile phone dependence has become increasingly prominent (Nikhita et al., 2015). As a typical behavioral addiction, mobile phone dependence not only possesses the core characteristics common to behavioral addictions, such as tolerance, withdrawal symptoms, and loss of control, but also exhibits unique manifestations including unbounded scenarios, strong addictive stickiness, concealment, and susceptibility to group diffusion due to the high convenience and multi-source reinforcement of smartphones (Mei et al., 2023; Sixto-Costoya et al., 2021; Yadav et al., 2021). Mobile phone dependence not only causes cervical spine problems and vision loss in adolescents (Fontenele et al., 2023; Özalp, 2025; Zhuang et al., 2021), but also has a negative impact on their mental health and social adaptability (Luo et al., 2025; Seo et al., 2016). At present, relevant studies (Chen et al., 2025; Li et al., 2023) have confirmed that regular physical exercise can reduce adolescents’ mobile phone dependence. Moreover, Liu et al. (2022) found that the effect of open-skill exercise intervention on improving college students’ mobile phone dependence is better than that of closed-skill exercise intervention. However, research on the correlation between exercise types and adolescent mobile phone dependence remains relatively scarce, with a lack of necessary empirical studies.

To address the aforementioned research gaps, this study aims to explore the correlations between exercise types, adolescents’ executive function, and mobile phone dependence from the perspective of motor skill classification, while testing the following research hypotheses:

*RH1*: Compared with adolescents engaging in closed-skill exercise, those participating in open-skill exercise demonstrate superior executive function.

*RH2*: Compared with adolescents engaging in closed-skill exercise, those participating in open-skill exercise exhibit lower levels of mobile phone dependence.

Through this study, it is anticipated to enrich the relevant theories in sport psychology and developmental psychology, and provide a new perspective for understanding the impact of physical exercise on adolescents’ cognitive and behavioral development. In addition, it aims to offer scientific guidance for adolescents in selecting appropriate exercise types, helping them improve executive function, reduce mobile phone dependence, and promote the comprehensive development of physical and mental health through participation in suitable physical exercise.

## Materials and methods

### Participants

This study utilized the G⋅Power 3.1.9.7 statistical software to calculate the sample size. Considering that the research aims to explore the impact of multiple factors on specific outcomes, a multiple regression model was ultimately chosen as the fundamental framework for the calculation. In terms of specific parameter settings, the effect size ES *f*^2^ was set to 0.15. This value corresponds to a medium effect level in statistics, which can well balance the sensitivity and practical operability of the study. Meanwhile, in this study, the significance level α was strictly set to 0.05, and the test power 1-β was set to 0.80. Combined with the 9 predictive factors involved in this study, the minimum required sample size for this research was finally determined to be 114 participants.

This study selected high school students in Sichuan Province for investigation. Firstly, according to literature reviews, high school students are still in the critical stage of executive function development. Secondly, Sichuan Province has actively explored and implemented the course-selective and class-moving teaching system. Under this system, students have long-term engagement in the learning and practice of a single sport, accumulating extensive experience. In addition, the physical exercise information of students under this teaching model is basically consistent. Specifically, they exercise twice a week for 45 min each time. Thirdly, from the perspective of the Trans theoretical Model (Abdoli et al., 2025; Sheng et al., 2023), 6 months is the standard time for behavior change, and behaviors tend to stabilize at the 6-month mark. That is, after 6 months of learning and practicing a certain program, students can acquire a relatively stable exercise behavior pattern from physical education classes. Finally, considering that senior three students are under great pressure to enter higher education, it is not appropriate to occupy too much of their study time. Therefore, first and second-year high school students with at least half a year of experience in specialized learning and practice were selected for the investigation.

In terms of sample selection, this study randomly selected 3 cities in Sichuan Province. In each selected city, the research team adopted a convenience sampling method to select 1 high school as the research school. In each research school, to ensure the rationality of the sample distribution across grades, the research team randomly selected 4 classes from Grade 10 and 4 classes from Grade 11 to participate in the research. It is worth noting that due to the enormous pressure of the college entrance examination and extremely heavy academic tasks faced by students in Grade 12, the research team decided not to include Grade 12 in this research to avoid interfering with their normal study. Through the above sampling process, this study involved a total of 24 classes and initially collected data from 1,278 students.

However, during the data sorting process, this study found that some questionnaires had incomplete responses, that is, some important questions were not answered, which would affect the completeness of the data and the accuracy of the analysis. In addition, some questionnaires were filled out unclearly with illegible handwriting, making it impossible to accurately interpret the students’ intentions. Moreover, there were a few cases where students filled out the questionnaires randomly, such as following a “1”—shaped or “Z”—shaped pattern, which obviously indicated that they did not take the research seriously. To ensure the quality of the data entering the data analysis stage, this study conducted a strict screening of all collected questionnaires and excluded the data of 162 students with the above-mentioned problems. After screening, the final valid sample included in this data analysis was 1,016 students. This study was conducted in strict accordance with the core principles and requirements of the Declaration of Helsinki, and has been reviewed and approved by the Scientific Research Ethics Committee of Sichuan University of Science & Engineering. All participants voluntarily signed written informed consent forms.

### Variables and data collection

#### Exercise types

In this study, students were surveyed via questionnaires regarding the sports programs they had recently been practicing and whether they had switched programs within the past 6 months. The surveyed students mainly participated in eight sports programs: football, basketball, volleyball, table tennis, badminton, track and field, martial arts, and aerobics. According to the classification criteria of motor skills (Zhang, 2012), football, basketball, volleyball, table tennis, and badminton belong to open skills; track and field, martial arts, and aerobics belong to closed skills. Notably, the reason why martial arts are classified as closed skills in this study is that in actual martial arts teaching practices, instructors primarily adopt routine-based drills for instruction, resulting in students lacking corresponding elements such as interpersonal interaction and complex environments during their learning and training processes. Based on this, the participants in this study were divided into two groups: those engaged in open skill exercises and those engaged in closed skill exercises.

#### Ecological executive function

Given the limitations of computer-based button-pressing tests for executive function in terms of ecological validity (Gioia et al., 2002), this study instead adopted a behavior rating scale that is more relevant to real-life scenarios for assessment. Specifically, the Adolescent Executive Function Scale (EFS-A), developed by Huang et al. (2014), was selected as the data collection tool to obtain information related to adolescents’ executive function. This questionnaire consists of 21 items and measures adolescents’ executive function from three dimensions: inhibitory control (6 items), working memory (7 items), and cognitive flexibility (8 items). In terms of scoring, the EFS-A uses a 3-point scale, where “1” represents “never,” “2” represents “sometimes,” and “3” represents “often.” The total score is calculated using a reverse scoring method; higher total scores indicate better performance in executive function (Wang, 2023). Results of reliability and validity tests show that the three dimensions of the questionnaire can collectively explain 45.39% of the variance, with factor loadings of each item ranging from 0.499 to 0.727. Meanwhile, the Cronbach’s α coefficient for the entire scale and its respective factors fall within the range of 0.786–0.897. These data indicate that the questionnaire has good reliability and validity, making it suitable for the measurement needs of this study.

#### Mobile phone dependence

To investigate the participants’ mobile phone dependence status, this study adopted the Self-Rated Questionnaire for Adolescent Mobile Phone Use Dependence developed by Tao et al. (2013). The questionnaire consists of 13 items, divided into three dimensions: withdrawal symptoms (6 items), craving (3 items), and physical and mental impact (4 items). The questionnaire uses a 5-point Likert self-rating scale for scoring, with scores ranging from 13 to 65. A higher score indicates a more severe level of mobile phone dependence. Tao et al. (2013) has verified the reliability of the questionnaire: the Cronbach’s α coefficient of the total questionnaire reaches 0.87, and the Cronbach’s α coefficient of each dimension range from 0.58 to 0.83, showing high internal consistency reliability, which is suitable for the measurement needs of this study.

#### Control variables

Drawing on relevant studies on the influencing factors of executive function (Lawson et al., 2018; Navayuth and Yurayat, 2022; Thasleema and Verma, 2024), this research selects variables such as age, gender, place of residence, body mass index (BMI), socio-economic status (SES), and parental educational level for control. Specifically, place of residence is categorized into two types: “urban” and “rural.” BMI values are calculated using the formula (BMI = kg/m^2^) based on height and weight. Adolescents’ SES is measured using the MacArthur Subjective Socio- economic Status Scale (Moss et al., 2023). This scale consists of a ladder diagram with 10 rungs and corresponding questions, where the top rung of the ladder represents the group with the highest SES, and the bottom rung represents the group with the lowest SES. Adolescents position themselves on a specific rung of the ladder according to their subjective feelings, thereby reflecting their subjective SES. Relevant studies (Amir et al., 2019; Sun et al., 2019) have confirmed that this scale can effectively assess the SES of respondents and is widely used in large-scale data surveys such as the China General Social Survey (CGSS). In addition, parental educational attainment was coded with reference to previous studies (Zhong and Wang, 2020; Zhong et al., 2020). Specifically, “no education received” and “primary school” were coded as 1 (i.e., primary school or below); “junior school” was coded as 2; and “technical secondary school,” “vocational high school,” “general high school,” “college diploma,” and “bachelor’s degree” were coded as 3 (i.e., senior school or above). The reason for coding “college diploma” and “bachelor’s degree” as 3 in this study was that the proportion of students whose parents held a college diploma or bachelor’s degree was relatively small.

#### Research procedures

Firstly, researchers were trained to familiarize themselves with the usage of research tools and the research procedures. Secondly, with the cooperation of schools, questionnaires and testing tools were distributed to adolescents, and data were collected through group testing. During the testing process, researchers provided unified explanations of the instructions for the questionnaires and tests to ensure that the participants understood the testing requirements. Finally, the questionnaires and test data were retrieved, and the data were sorted out and screened to eliminate invalid data.

#### Data processing and statistical analysis

In this study, in accordance with specific research needs, SPSS 21.0 software were used for data processing and statistical analysis. For continuous variables, descriptive statistics were conducted using mean (*M*) and standard deviation (*SD*); for categorical variables, descriptive statistics were performed using frequency and percentage. Firstly, the normality of data on ecological executive function and mobile phone dependence variables was tested by means of the one-sample Kolmogorov-Smirnov test combined with P-P plots and Q-Q plots. The results showed that the data approximately presented a normal distribution. Therefore, independent samples *t*-test and One-Way ANOVA were adopted to conduct inter-group comparative analysis based on classification data of basic information such as gender, place of residence, and parental educational level. Secondly, this study adopted the linear response model of the generalized linear model (GLM), with ecological executive function and mobile phone dependence as the dependent variables and exercise types as the independent variable, to explore the correlations between exercise types and ecological executive function, as well as between exercise types and mobile phone dependence, after controlling for relevant confounding factors. Finally, partial correlation analysis was used to explore the correlation between ecological executive function and mobile phone dependence. On this basis, this study took exercise type as the independent variable, mobile phone dependence as the dependent variable, and ecological executive function as the mediating variable to explore the potential mediating effect of ecological executive function in the relationship between exercise type and mobile phone dependence. The test level for all the above statistical methods was defined as α = 0.05.

## Results

### Basic information of participants

A total of 1,016 adolescents were included, with an average age of (16.14 ± 0.57) years. Among them, 60.6% were female, 75.6% were rural students, and 56.7% were adolescents who participated in open-skill exercises. In addition, among the 1,016 included adolescents, the majority of fathers had an educational level of junior school (51.6%), while the majority of mothers had educational levels of junior school (40.9%) and primary school or below (41.7%). The adolescents’ inhibitory control (13.88 ± 2.66), working memory (15.33 ± 3.07), and cognitive flexibility (17.01 ± 3.28) were roughly at a medium to upper level; withdrawal symptoms (13.25 ± 5.63), craving (6.72 ± 2.69), and physical and mental impacts (9.64 ± 3.75) were at a medium to lower level. Moreover, the average BMI of the included adolescents was (21.60 ± 4.99) kg/m^2^, which is within the normal range; the average SES was at a medium level (5.79 ± 0.91). The basic information of the study subjects is detailed in Table 1.

**TABLE 1:** The basic information of the participants.

Continuous variables	*M*	*SD*	Min	Max	Categorical variables	Freq.	%
Age (years)	16.14	0.57	15	18	Gender	400	39.4
BMI (kg/m^2^)	21.60	4.99	15.24	44.82	Boys		
SES (score)	5.79	0.91	2	10	Girls	616	60.6
Inhibitory control (score)	13.88	2.66	6.00	18.00	Residence	248	24.4
Working memory (score)	15.33	3.07	7.00	21.00	Urban		
Cognitive flexibility (score)	17.01	3.28	8.00	24.00	Rural	768	75.6
Withdrawal symptoms (score)	13.25	5.63	6.00	30.00	father’s educational attainment		
Craving (score)	6.72	2.69	3.00	15.00	Primary school and below	256	25.2
Physical and mental impacts (score)	9.64	3.75	4.00	20.00	Junior school	524	51.6
Categorical variables	Freq.	%			Senior school and above	236	23.2
Exercise types					mother’s educational attainment		
Open skills	576	56.7			Primary school and below	424	41.7
Closed skills	440	43.3			Junior school	416	40.9
					Senior school and above	176	17.3

BMI, body mass index; SES, socioeconomic status.

### Comparative analysis of executive function and mobile phone dependence between groups based on gender, residence, and parental educational attainment

**FIGURE 1:**
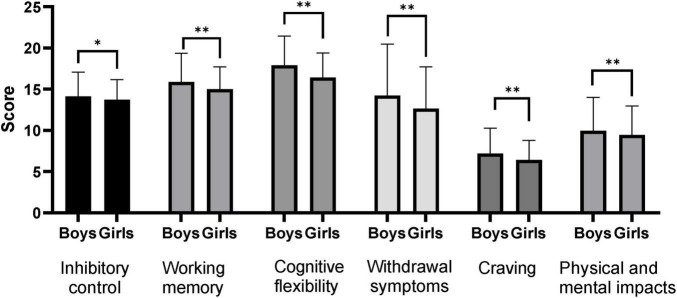
Comparative analysis of adolescents’ executive function and mobile phone dependence between groups based on gender. *P < 0.05; **P < 0.01.

**FIGURE 2:**
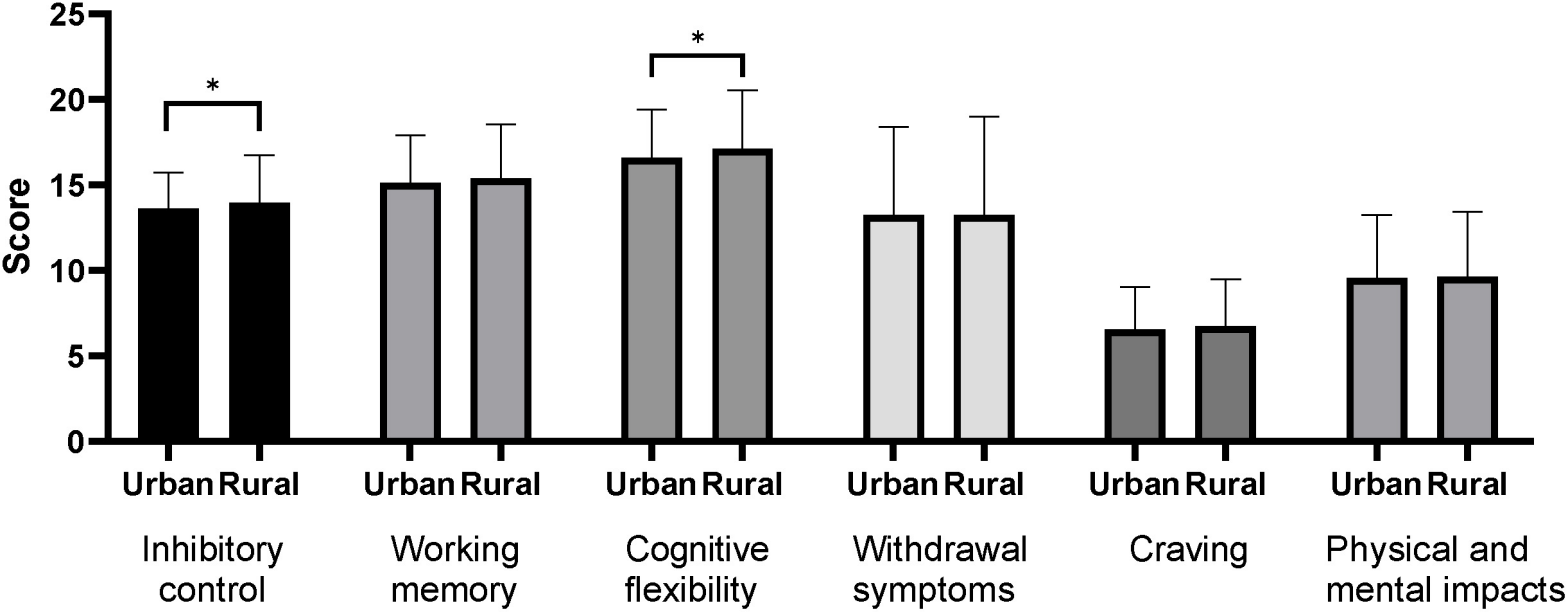
Comparative analysis of adolescents’ executive function and mobile phone dependence between groups based on residence. *P < 0.05.

Results of the gender-based between-group difference analysis (Figure 1) showed that boys had significantly higher levels of executive function (inhibitory control, working memory, cognitive flexibility) and mobile phone dependence (withdrawal symptoms, craving levels, physical and mental health impacts) than girls (*P* < 0.05). Results of the between-group difference analysis based on place of residence (Figure 2) showed that rural adolescents had significantly higher inhibitory control and cognitive flexibility than urban adolescents (*P* < 0.05), while there were no significant differences between rural and urban adolescents in working memory and mobile phone dependence (withdrawal symptoms, craving levels, and physical and mental health impacts) (*P* > 0.05).

**FIGURE 3:**
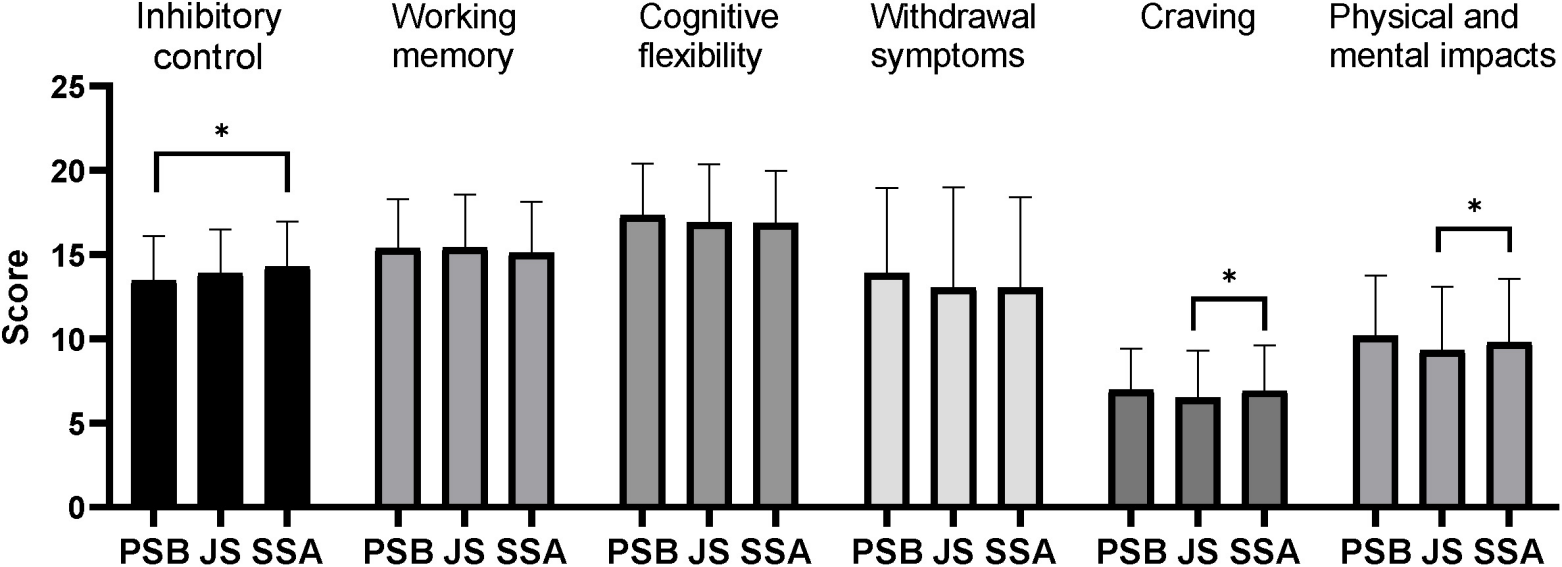
Comparative analysis of adolescents’ executive function and mobile phone dependence between groups based on father’s educational attainment. PSB, primary school and below; JS, junior school; SSA, senior school and above. *P < 0.05.

**FIGURE 4:**
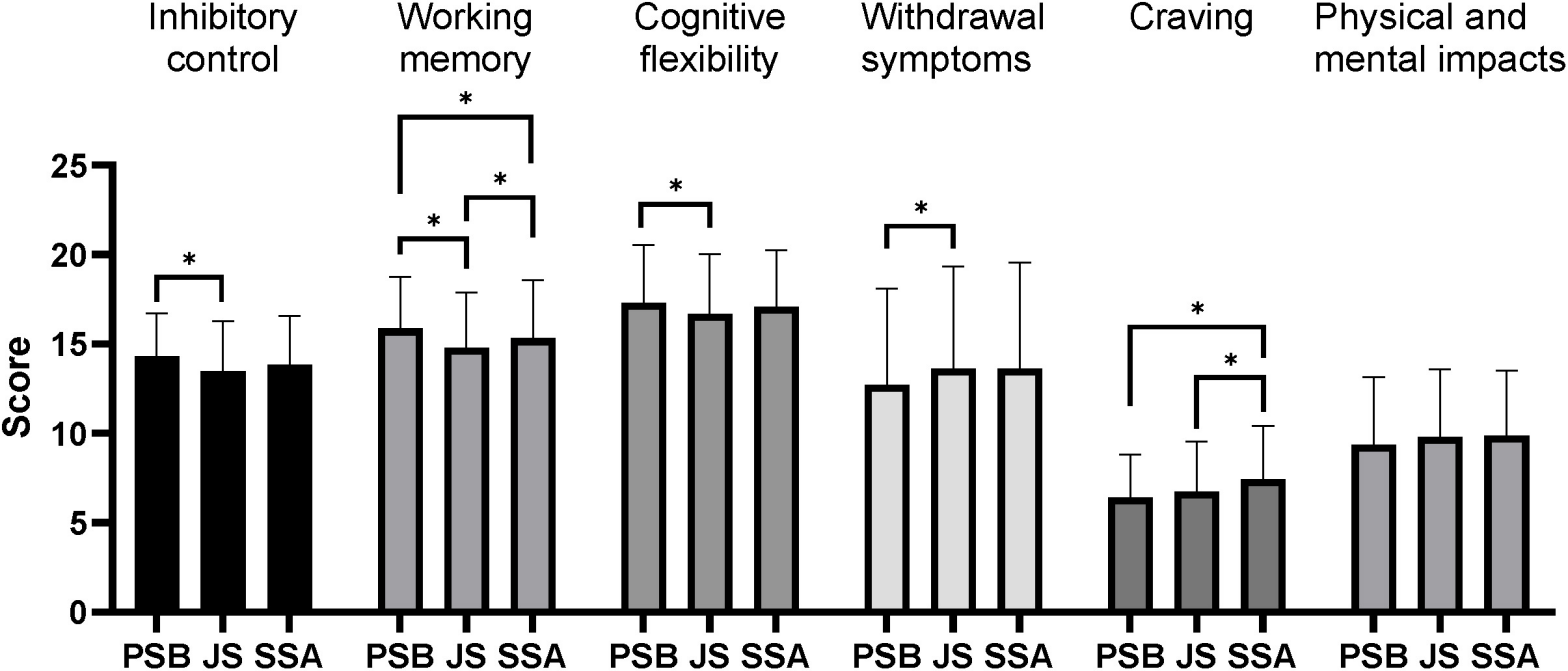
Comparative analysis of adolescents’ executive function and mobile phone dependence between groups based on mother’s educational attainment. PSB, primary school and below; JS, junior school; SSA, senior school and above. *P < 0.05.

Results of the between-group difference analysis based on the father’s educational level (Figure 3) showed that adolescents whose fathers had an educational level of primary school or below had significantly lower inhibitory control ability than those whose fathers had an educational level of senior high school or above (*P* < 0.05), and their craving for mobile phone dependence and the impacts on physical and mental health were significantly higher than those whose fathers had an educational level of junior high school (*P* < 0.05). The results of the inter-group comparative analysis based on mother’s educational level (Figure 4) showed that adolescents whose mothers have an educational level of primary school or below have significantly higher inhibitory control than those whose mothers have a junior school education (*P* < 0.05), significantly higher working memory than those whose mothers have an educational level of junior school or above (*P* < 0.05), significantly higher cognitive flexibility than those whose mothers have a junior school education (*P* < 0.05), significantly lower withdrawal symptoms of mobile phone dependence than those whose mothers have a junior school education (*P* < 0.05), and significantly lower craving for mobile phone dependence than those whose mothers have an educational level of senior school or above (*P* < 0.05); adolescents whose mothers have a junior school education have significantly lower working memory and craving for mobile phone dependence than those whose mothers have an educational level of senior school or above (*P* < 0.05).

### The correlation between exercise types and executive function

The results of the multiple linear regression analysis on exercise types and adolescents’ executive function (Table 2) showed that compared with adolescents engaging in closed-skill exercises, those participating in open-skill exercises had higher inhibitory control (β = 0.410, 95%CI = 0.083∼0.738, Wald χ^2^= 6.026, *P* = 0.014) and cognitive flexibility (β = 0.588, 95%CI = 0.188∼0.988, Wald χ^2^= 8.305, *P* = 0.004). However, there was no significant difference in working memory between the two groups of adolescents (β = 0.257, 95%CI = −0.124∼0.638, Wald χ^2^= 1.751, *P* = 0.186).

**TABLE 2:** Results of multiple linear regression analysis on the correlation between exercise types and adolescents’ executive function.

Variables	Inhibitory control			Working memory			Cognitive flexibility		
	β	95%CI	*P*	β	95%CI	*P*	β	95%CI	*P*
**Exercise types**									
OS	0.410	0.083, 0.738	0.014	0.257	−0.124, 0.638	0.186	0.588	0.188, 0.988	0.004
Age	0.100	−0.180, 0.381	0.483	0.016	−0.310, 0.342	0.921	0.821	0.478, 1.164	0.000
**Gender**									
Boys	0.435	0.101, 0.768	0.011	0.918	0.531, 1.306	0.000	1.311	0.903, 1.718	0.000
**Residence**									
Urban	−0.531	−0.916, −0.146	0.007	−0.141	−0.588, 0.306	0.536	−0.358	−0.828, 0.112	0.135
**Father’s educational attainment**									
Primary school and below	−1.126	−1.613, −0.640	0.000	0.102	−0.463, 0.668	0.723	0.208	−0.387, 0.802	0.493
Junior school	−0.631	−1.058, −0.204	0.004	0.333	−0.163, 0.829	0.188	−0.145	−0.666, 0.376	0.585
**Mother’s educational attainment**									
Primary school and below	0.683	0.202, 1.164	0.005	0.376	−0.183, 0.934	0.188	0.037	−0.551, 0.624	0.902
Junior school	−0.304	−0.791, 0.183	0.221	−0.746	−1.311, −0.180	0.010	−0.577	−1.172, 0.017	0.057
BMI	−0.003	−0.036, 0.029	0.839	−0.058	−0.095, −0.021	0.002	−0.028	−0.068, 0.011	0.156
SES	0.414	0.229, 0.598	0.000	0.239	0.025, 0.453	0.029	0.232	0.007, 0.457	0.044

OS, open skills; BMI, body mass index; SES, socioeconomic status.

Age was significantly positively correlated with cognitive flexibility (*P* = 0.000). Compared with girls, boys had higher inhibitory control, working memory, and cognitive flexibility (*P*< 0.05). Compared with rural adolescents, urban adolescents had lower inhibitory control (*P* = 0.007). Compared with adolescents whose fathers had an education level of senior high or above, those whose fathers had an education level of junior school or below had lower inhibitory control (*P*< 0.01). Compared with adolescents whose mothers had an education level of senior school or above, those whose mothers had an education level of primary school or below had lower inhibitory control (*P* = 0.005), and those whose mothers had an education level of junior school had lower working memory (*P* = 0.010). BMI was significantly negatively correlated with working memory (*P* = 0.002). SES was significantly positively correlated with inhibitory control, working memory, and cognitive flexibility (*P*< 0.05). Detailed results are shown in Table 2.

### The correlation between exercise types and mobile phone dependence

The results of the multiple linear regression analysis on exercise types and adolescents’ mobile phone dependence (Table 3) showed that there were no significant differences in withdrawal symptoms (β = 0.390, 95%CI = −0.314∼1.093, Wald χ^2^= 1.177, *P* = 0.278) and craving (β = 0.037, 95%CI = −0.299∼0.372, Wald χ^2^= 0.046, *P* = 0.831) between adolescents engaging in open-skill exercises and those participating in closed-skill exercises. However, compared with adolescents taking part in closed-skill exercises, those doing open-skill exercises had lower physical and mental impacts (β = −0.600, 95%CI = −1.073∼-0.127, Wald χ^2^= 6.174, *P* = 0.013).

**TABLE 3:** Results of multiple linear regression analysis on the correlation between exercise types and adolescents’ mobile phone dependence.

Variables	Withdrawal symptoms			Craving			Physical and mental impacts		
	β	95%CI	*P*	β	95%CI	*P*	β	95%CI	*P*
**Exercise types**									
OS	0.390	−0.314, 1.093	0.278	0.037	−0.299, 0.372	0.831	−0.600	−1.073, −0.127	0.013
Age	1.031	0.428, 1.633	0.001	0.365	0.077, 0.652	0.013	0.234	−0.171, 0.639	0.257
**Gender**									
Boys	1.486	0.770, 2.203	0.000	0.635	0.293, 0.977	0.000	0.635	0.154, 1.117	0.010
**Residence**									
Urban	0.215	−0.611, 1.042	0.610	−0.237	−0.631, 0.157	0.239	−0.206	−0.761, 0.527	0.468
**Father’s educational attainment**									
Primary school and below	0.995	−0.050, 2.041	0.062	0.198	−0.301, 0.607	0.436	0.492	−0.211, 1.195	0.170
Junior school	−0.116	−1.033, 0.801	0.804	−0.250	−0.687, 0.188	0.263	−0.506	−1.123, 2.593	0.107
**Mother’s educational attainment**									
Primary school and below	−1.141	−2.174, −0.108	0.030	−1.060	−1.553, −0.567	0.000	−0.534	−1.229, 0.161	0.132
Junior school	−0.164	−1.209, 0.882	0.759	−0.660	−1.158, −0.161	0.010	−0.100	−0.802, 0.603	0.781
BMI	−0.035	−0.104, 0.034	0.317	0.014	−0.019, 0.047	0.399	0.015	−0.031, 0.062	0.514
SES	0.310	−0.086, 0.706	0.125	−0.162	−0.351, 0.027	0.093	0.281	0.015, 0.547	0.039

OS, open skills; BMI, body mass index; SES, socioeconomic status.

Age was significantly positively correlated with withdrawal symptoms and craving (*P*< 0.05). Compared with girls, boys had higher withdrawal symptoms, craving, and physical and mental impacts (*P*< 0.05). Compared with adolescents whose mothers had an education level of senior school or above, those whose mothers had an education level of primary school or below had lower withdrawal symptoms and craving (*P*< 0.05), and those whose mothers had an education level of junior school had lower craving (*P*< 0.05). SES was significantly positively correlated with physical and mental impacts (*P*< 0.05). In addition, there was no significant difference in mobile phone dependence between urban and rural adolescents (*P*> 0.05); there was no significant difference in mobile phone dependence among adolescents with different fathers’ education levels (*P*> 0.05); the correlation between BMI and mobile phone dependence was not significant (*P*> 0.05) (for specific results, see Table 3 for details).

### The mediating effect of executive function on the relationship between exercise types and mobile phone dependence

The results of the partial correlation analysis between adolescents’ executive function and mobile phone dependence (Table 4) showed that inhibitory control was significantly negatively correlated with withdrawal symptoms (*r* = −0.337), craving (*r* = −0.300), and physical and mental impact (*r* = −0.297) (*P*< 0.01); working memory was significantly negatively correlated with withdrawal symptoms (*r* = −0.303), craving (*r* = −0.269), and physical and mental impact (*r* = −0.292) (*P*< 0.01); cognitive flexibility was significantly negatively correlated with withdrawal symptoms (*r* = −0.347), craving (*r* = −0.288), and physical and mental impact (*r* = −0.397) (*P*< 0.01). In conclusion, there was a significant negative correlation between adolescents’ executive function and mobile phone dependence (*P*< 0.01).

**TABLE 4:** Results of partial correlation analysis between adolescents’ executive function and mobile phone dependence.

Variables	Inhibitory control	Working memory	Cognitive flexibility	Withdrawal symptoms	Craving	Physical and mental impacts
Inhibitory control	1.000	0.559**	0.616**	−0.337**	−0.300**	−0.297**
Working memory	0.559**	1.000	0.583**	−0.303**	−0.269**	−0.292**
Cognitive flexibility	0.616**	0.583**	1.000	−0.347**	−0.288**	−0.397**
Withdrawal symptoms	−0.337**	−0.303**	−0.347**	1.000	0.805**	0.791**
Craving	−0.300**	−0.269**	−0.288**	0.805**	1.000	0.721**
Physical and mental impacts	−0.297**	−0.292**	−0.397**	0.791**	0.721**	1.000

**P<0.01.

**FIGURE 5:**
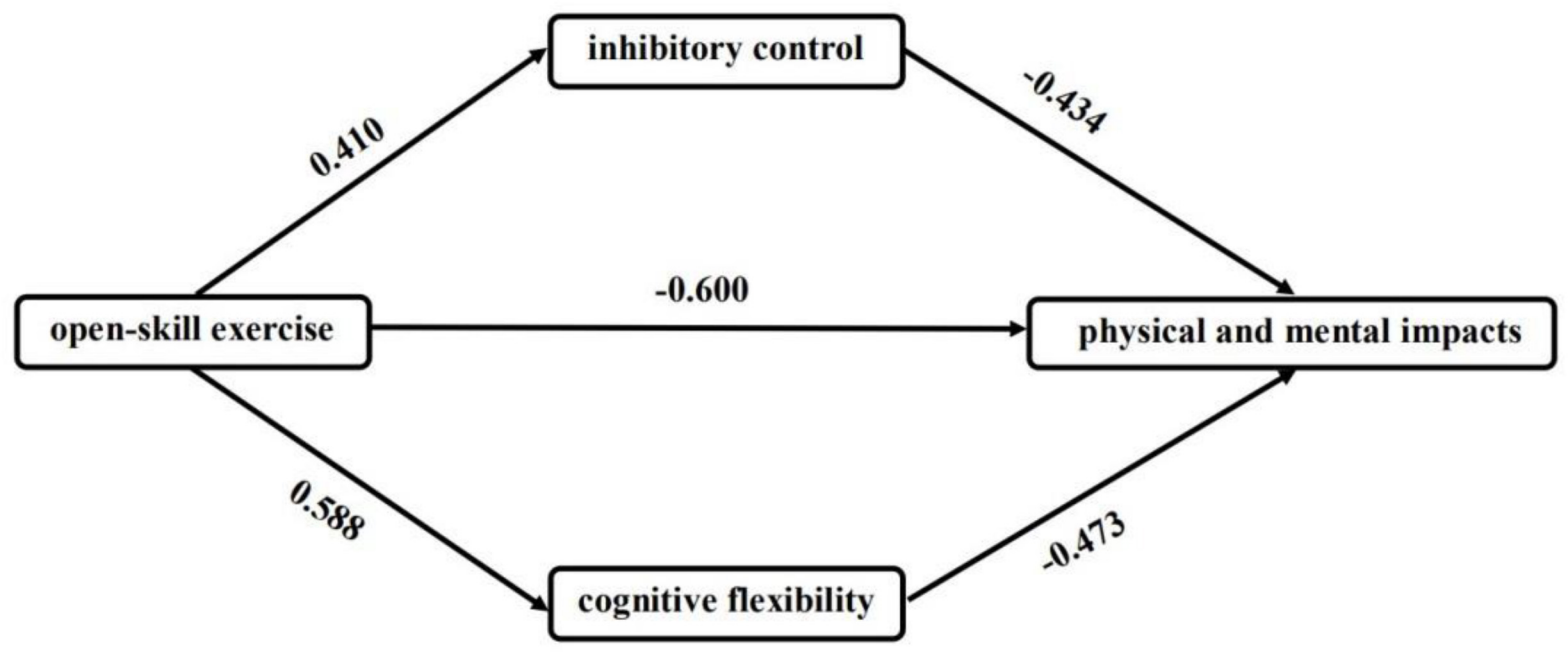
Mediating effect diagram.

Based on the results in Tables 2, 3, it was found that open-skill exercise was significantly positively correlated with inhibitory control (β = 0.410, *P* = 0.014) and cognitive flexibility (β = 0.588, *P* = 0.004); open-skill exercise was significantly negatively correlated with physical and mental impact (β = −0.600, *P* = 0.013). In addition, inhibitory control and cognitive flexibility were significantly negatively correlated with the physical and mental impact caused by mobile phone dependence (*P*< 0.01). Therefore, inhibitory control and cognitive flexibility may have potential mediating roles in the relationship between open-skill exercise and physical and mental impact. Further mediating effect test (Table 5) showed that the direct effect of open-skill exercise on physical and mental impact was −0.600, the effect of open-skill exercise on inhibitory control was 0.410, and the direct effect of inhibitory control on physical and mental impact was −0.434. Thus, the indirect effect of inhibitory control in the influence of open-skill exercise on physical and mental impact was 0.410 × (−0.434) = −0.178; the effect of open-skill exercise on cognitive flexibility was 0.588, and the direct effect of cognitive flexibility on physical and mental impact was −0.473. Therefore, the indirect effect of cognitive flexibility in the influence of open-skill exercise on physical and mental impact was 0.588 × (−0.473) = −0.278. The mediating effect is detailed in Figure 5.

**TABLE 5:** Results of testing the mediating role of inhibitory control and cognitive flexibility in the relationship between open-skill exercise and physical and mental impacts.

Variables	Physical and mental impacts			Inhibitory control	Physical and mental impacts			Cognitive flexibility
OS	−0.600*	−0.434**	−0.424*	0.410*	−0.600*	−0.477**	−0.322	0.588*
Inhibitory control			−0.428**				−0.473**	
Cognitive flexibility								
Control variables	Control	Control	Control	Control	Control	Control	Control	Control

OS, open skills. *P< 0.05; **P < 0.01.

## Discussion

### Groups engaging in open-skill exercises have higher levels of inhibitory control and cognitive flexibility

The results of this study found that adolescents engaged in open-skill exercises have higher inhibitory control and cognitive flexibility compared with those participating in closed-skill exercises. This is consistent with the conclusions of many previous studies based on computerized test tasks (Feng et al., 2023; Shi et al., 2022). It is closely related to the enriched environment, high-frequency interpersonal interaction and complex task challenges during the exercise process (Shi and Feng, 2022; Shi et al., 2023).

The dynamic and unpredictable scenarios of open-skill exercises drive individuals to form more efficient cognitive regulation patterns (Zhang, 2012). During exercise, participants are required to capture multi-dimensional information such as opponents’ movements and venue changes in real time. This process continuously activates the brain’s adaptive cognitive system, prompting individuals to constantly optimize cognitive strategies such as information screening and goal priority ranking (Alister et al., 2024; Raichlen and Alexander, 2017). The cognitive challenges brought by open-skill exercises can enhance the brain’s efficiency in processing dynamic information, thereby improving executive functions (Li Q. et al., 2024; Zhou et al., 2020). In addition, open-skill exercises are mostly team-based or confrontational activities with intensive and complex interpersonal interactions, and such social scenarios have much higher cognitive requirements than the independent completion mode of closed-skill exercises (Shi et al., 2023; Zhang, 2012). Meanwhile, the tasks of open-skill exercises are multi-objective with dynamic rules, requiring adolescents to handle multiple cognitive subtasks simultaneously. This complexity directly challenges the cognitive regulation system (Shi et al., 2023; Shi and Feng, 2022). Furthermore, the cognitive benefits of open-skill exercises are transferable across scenarios (Heilmann et al., 2022). Most open-skill exercises are team-based or competitive events that require cognitive decision-making in real-world social and task contexts. Unlike the single-dimensional measurements of standardized laboratory tasks, such cognitive training in authentic and complex ecological scenarios can directly enhance adolescents’ ability to apply executive functions in their daily study and life (Pasqualotto et al., 2021), such as planning learning tasks more efficiently, responding flexibly to unexpected classroom situations, and rationally controlling attention to avoid being distracted by irrelevant things. In contrast, the environment of closed-skill exercises is stable, with single and predictable information. They have low demands for information screening and cognitive reorganization, minimal needs for multi-task processing and rule switching, and only require focusing on one’s own state. They lack the need for “inferring others’ intentions” and “dynamically adjusting strategies,” thus having a weaker stimulation effect on inhibitory control and cognitive flexibility. In conclusion, open-skill exercises provide a continuous and high-intensity training scenario for adolescents’ inhibitory control (filtering out irrelevant information and suppressing impulses) and cognitive flexibility (adapting to new environments and switching strategies) through information screening in dynamic environments, social cognitive challenges in high-frequency interpersonal interactions, and multi-objective regulation in complex tasks.

However, this study did not find a significant difference in working memory between the two types of exercises, which is inconsistent with the results of some previous studies (Shi et al., 2023; Zhang, 2012). A possible reason is that the EFS-A used in this study focuses on ecological executive function and emphasizes the application of working memory in daily scenarios (such as remembering task arrangements and planning study schedules), rather than specialized memory tasks like the 2-back task in laboratories. Secondly, open-skill exercises may only improve working memory specific to sports scenarios, but their transfer effect on daily working memory is limited, thus no inter-group difference was shown. In the sports scenarios of open skills, adolescents need to process a large amount of dynamic information in real time and maintain memory. The improvement of this “sports-specific working memory” is to meet the immediate needs of sports scenarios. This immediacy requirement is consistent with the test requirements of specialized memory tasks such as the 2-back task, but it is vastly different from the requirements of ecological action memory.

### Groups engaging in open-skill exercises have lower levels of physical and mental impacts

The results of this study found that adolescents participating in open-skill exercises have lower physical and mental impacts caused by mobile phone dependence compared with those engaging in closed-skill exercises. This study result partially supports the conclusion of Liu et al. (2022) that open-skill exercises are more effective in improving college students’ mobile phone dependence. This outcome may be closely related to the unique attributes of open-skill exercises and their influence on adolescents’ behavioral patterns and mental states.

Firstly, open-skill exercises require participants to continuously focus on multi-dimensional information in a dynamic environment, a process that demands a high degree of concentration and the ability to quickly screen information (Monsma et al., 2017). Long-term participation in such exercises can strengthen adolescents’ active attention regulation ability, making it easier for them to get rid of passive attention dependence on mobile phones (such as meaningless scrolling through short videos, frequent checking of social information, etc.) (Monsma et al., 2017; Zhou et al., 2020). In contrast, closed-skill exercises take place in a stable environment with fixed processes, and have lower requirements for dynamic regulation of attention, so they are less effective in improving the problem of attention consolidation caused by mobile phone dependence. Secondly, open-skill exercises are mostly team-based or confrontational activities, during which adolescents engage in frequent and in-depth interpersonal interactions, such as collaborative coordination, strategic communication, and emotional resonance. Such real social scenarios can meet adolescents’ psychological needs such as sense of belonging and recognition. One of the major inducements of mobile phone dependence is the excessive reliance on virtual social interactions (such as online chatting and social media interactions) (Billieux, 2012). When open-skill exercises provide richer social experiences with more immediate feedback, adolescents’ demand for mobile phone-based socializing will naturally decrease, thereby reducing the physical and mental impacts such as loneliness and anxiety caused by excessive use of mobile phones. Finally, some adolescents escape from real-life pressures and alleviate negative emotions by scrolling through their mobile phones (Hoffner and Lee, 2015). In open-skill exercises, adolescents need to deal with challenges such as unexpected situations in competitions and conflicts within the team, which forces them to quickly adjust their emotions and stay calm under pressure. Long-term participation in such exercises can enhance the flexibility of their emotional regulation (Bernstein and McNally, 2018).

However, this study found that there was no significant difference in withdrawal symptoms and craving between the groups engaging in open-skill exercises and closed-skill exercises. The reasons for this can be analyzed as follows: Firstly, cross-sectional studies may not be able to observe the corresponding effects; secondly, behavioral habits such as withdrawal symptoms and craving are highly stable and require long-term exercise intervention to change; while physical and mental impacts are more sensitive to short-term behavioral adjustments, thus showing significant differences in cross-sectional data.

### The mediating role of inhibitory control and cognitive flexibility in the relationship between open-skill exercise and physical and mental impacts

The results of this study revealed a significant negative correlation between executive function and mobile phone dependence, indicating that the stronger the executive function, the lower the level of mobile phone dependence. Further mediating effect tests confirmed that inhibitory control and cognitive flexibility play a partial mediating role in the relationship between open-skill exercises and physical and mental impacts. That is, open-skill exercises can reduce the physical and mental harm caused by mobile phone use by improving inhibitory control and cognitive flexibility.

In this study, the potential reasons why open-skill exercises are more conducive to the improvement of inhibitory control and cognitive flexibility have been explained in the above discussion, and will not be elaborated on here. Relevant studies (Ding et al., 2018; Yuan et al., 2023) have also confirmed that there is a significant negative correlation between inhibitory control, cognitive flexibility and mobile phone dependence. The improvement of inhibitory control can help individuals actively reduce excessive mobile phone use (such as setting and adhering to usage duration limits), thereby alleviating problems such as insufficient sleep and fragmented attention caused by uncontrolled phone scrolling, and thus reducing the physical and mental impacts of mobile phone dependence. In addition, the enhancement of cognitive flexibility enables individuals to plan mobile phone usage scenarios more rationally (for example, using phones only during rest periods instead of frequently getting distracted during work/study). This avoids conflicts between mobile phone use and other important life tasks, thereby reducing anxiety and impairment of real-life functions caused by the intrusion of mobile phones into daily life, and further mitigating the physical and mental effects of mobile phone dependence.

In conclusion, open-skill exercises are like a “gym for the brain.” By repeatedly training individuals’ abilities to “resist temptation” (inhibitory control) and “switch flexibly” (cognitive flexibility), they enable people to be more “rational” when faced with mobile phones, ultimately reducing the negative physical and mental impacts of mobile phone dependence. This provides a theoretical basis for improving adolescents’ mobile phone dependence through exercise intervention, that is, choosing open-skill exercises (such as basketball and badminton) can simultaneously enhance executive function and alleviate mobile phone dependence, achieving the coordinated development of physical and mental health.

### Discussion on inter-group comparisons of executive function by gender, residence, and parental education level

Firstly, in terms of gender, boys exhibited significantly higher executive function than girls, which is consistent with the findings of previous studies (Gaillard et al., 2021; Wu et al., 2024). On the one hand, there are gender-specific differences in the brain development processes of boys and girls. The developmental rhythm and functional activation patterns of the prefrontal lobe (closely associated with executive function) in boys differ from those in girls, which may underpin their superior cognitive regulation abilities (Chaku and Hoyt, 2019). On the other hand, in traditional educational contexts, boys are more likely to participate in competitive and strategic activities (e.g., ball games). Such activities can continuously train inhibitory control and cognitive flexibility, thereby indirectly enhancing overall executive function (Deaner et al., 2012).

Secondly, regarding residence, rural adolescents had significantly higher inhibitory control and cognitive flexibility than urban adolescents. This result is inconsistent with the findings of Li Y. et al. (2024), which suggested that urban adolescents outperformed rural counterparts in executive function. The participants in this study were high school students in Sichuan Province under the course-selective and class-moving teaching system, whose sports scenarios featured strong uniformity. Additionally, rural adolescents regularly engaged in unstructured activities such as farm work and group outdoor games in daily life. These activities required real-time environmental judgment and behavioral regulation, directly training ecological inhibitory control (e.g., filtering out irrelevant distractions) and cognitive flexibility (e.g., responding to unexpected situations). Furthermore, the cognitive stimulation for urban adolescents mostly came from stylized scenarios (e.g., classroom teaching, online learning), which provided insufficient training for ecological executive function. In contrast, rural adolescents’ cognitive stimulation originated from real and dynamic natural/social scenarios (e.g., coordinating farm work division, addressing outdoor emergencies), which were more aligned with the daily cognitive application abilities assessed by the EFS-A.

Thirdly, in terms of parental education level, adolescents whose fathers had an education level of primary school or below showed significantly lower inhibitory control than those whose fathers had a senior high school education or above; adolescents whose mothers had an education level of primary school or below demonstrated significantly higher executive function than those whose mothers had a junior high school education; and adolescents whose mothers had a junior high school education had significantly lower working memory than those whose mothers had a senior high school education or above. Parental education level exerts an impact through family parenting styles and cognitive environments. Although highly educated parents can provide richer cognitive stimulation, families where mothers have a higher education level may impose stricter academic requirements on their children, which is prone to causing excessive cognitive pressure on children and instead restricting the development of executive function. In contrast, in families where mothers have an education level of primary school or below, children have more opportunities to make independent decisions and solve problems, enabling them to improve inhibitory control and cognitive flexibility through practice. Fathers with higher education levels are more likely to establish a rational cognitive guidance environment for their children, facilitating the development of inhibitory control.

### Discussion on inter-group comparisons of mobile phone dependence by gender, residence, and parental education level

Firstly, in terms of gender, boys exhibited a more severe level of mobile phone dependence, which is consistent with the findings of previous studies (He and Huang, 2024; Yu et al., 2024). The main reasons are as follows: boys have a higher level of engagement in mobile games and competitive applications (Khan et al., 2017), and such applications are prone to triggering frequent usage and addictive tendencies. In addition, boys generally have relatively weak self-discipline awareness and emotional regulation abilities (Bear and Soltys, 2020), making it more difficult for them to control the duration and frequency of mobile phone use, and thus exhibiting more severe mobile phone dependence and associated physical and mental impairments.

Secondly, regarding residence, there was no significant difference in mobile phone dependence between urban and rural adolescents. With the popularization of digitalization in both urban and rural areas, the access channels, usage scenarios, and online content accessed by adolescents in urban and rural regions have gradually become similar. The inducing factors of mobile phone dependence (such as social needs and entertainment needs) do not show obvious regional differences, resulting in no observable difference in dependence levels between the two groups.

Finally, in terms of parental education level: adolescents whose fathers had an education level of primary school or below had significantly higher scores in craving for mobile phones and physical and mental impacts of dependence than those whose fathers had a junior high school education; adolescents whose mothers had an education level of primary school or below had significantly lower scores in withdrawal symptoms than those whose mothers had a junior high school education, and significantly lower craving scores than those whose mothers had a senior high school education or above; adolescents whose mothers had a junior high school education had significantly lower craving scores than those whose mothers had a senior high school education or above. Parental education level is related to the intensity of family supervision and communication patterns. Highly educated parents set clearer rules for their children’s mobile phone use, which can effectively reduce their children’s craving for mobile phones; in contrast, parents with lower education levels may adopt extensive supervision methods, making it difficult to guide their children to use mobile phones rationally, leading to higher scores in certain dimensions of mobile phone dependence. Meanwhile, as the primary caregivers, mothers’ educational backgrounds have a more direct impact on their children’s behavioral habits than fathers’, which is why inter-group differences in mobile phone dependence are more pronounced across maternal education levels.

### The value and significance of this study

From the perspective of motor skill classification (open skills vs. closed skills), this study systematically explored the correlation between different types of exercises and adolescents’ executive function and mobile phone dependence. It was the first to clarify the mediating mechanism through which open-skill exercises indirectly reduce the physical and mental impacts caused by mobile phone dependence by improving inhibitory control and cognitive flexibility. This finding provides a new theoretical explanatory framework for the cross-domain correlation of “exercise- cognition- behavior” and fills the research gap in existing studies regarding the association between ecological executive function (rather than laboratory-standardized tasks) and real-life behaviors (such as mobile phone use). In addition, this study offers a new perspective for analyzing the cognitive causes of adolescents’ mobile phone dependence, that is, mobile phone dependence is not only a matter of behavioral habits but also closely related to individuals’ cognitive regulation ability, thereby enriching the theoretical basis for interventions on mobile phone dependence.

This study provides scientific guidance for adolescents in choosing exercise types. It is found that open-skill exercises are more effective than closed-skill exercises in improving inhibitory control and cognitive flexibility, and can significantly reduce the physical and mental impacts of mobile phone dependence. This offers specific basis for adolescents, parents and schools in selecting sports programs, and it is suggested that open-skill exercises should be prioritized to achieve the dual benefits of physical fitness and cognitive improvement. In addition, this study provides a new strategy for the intervention of mobile phone dependence. Based on the research conclusions, an intervention program centered on open-skill exercises can be designed. Through systematic sports training, adolescents’ self-regulation ability (such as resisting the temptation of mobile phones and rationally planning usage scenarios) can be enhanced, replacing traditional passive intervention methods like “prohibiting use.” This active intervention mode, which focuses on improving cognitive ability, is more acceptable to adolescents and has a more lasting effect. Finally, this study can provide a basis for public health departments to formulate adolescent health policies. For example, by promoting open-skill sports programs, the problem of adolescent mobile phone dependence can be alleviated, and their coordinated physical and mental development can be promoted.

In summary, this study not only promotes the cross-integration of sports, cognition and behavior fields in theory, but also provides operable strategies for the healthy growth of adolescents (including cognitive development and behavior management) in practice, thus having important academic value and application prospects.

### Limitations of this study

Although this study found that adolescents engaging in open-skill exercises had higher inhibitory control and lower mobile phone dependence compared with those participating in closed-skill exercises, and also verified the mediating effect of inhibitory control and cognitive flexibility in the process of open-skill exercises alleviating the physical and mental impacts caused by mobile phone dependence, the study still has limitations in terms of research design, sample representativeness, data collection methods, executive function assessment dimensions, and potential confounding factor control.

Firstly, at the level of research design, this study adopted a cross-sectional design, which can only reveal the correlation between exercise types, executive function, and mobile phone dependence, but cannot determine the causal relationship among these variables. Moreover, due to the lack of longitudinal tracking data, it is impossible to clarify the direction of influence between variables. In addition, the study underestimates the interpretative limitations of this design and fails to fully illustrate its restrictions on external validity: cross-sectional studies can only reflect group characteristics at specific time points, making it difficult to generalize the findings to adolescent groups at different time stages. They also cannot capture the dynamic changes in exercise behavior, cognitive ability, and mobile phone usage behavior over time, and thus cannot provide evidence for the long-term effects of intervention programs. Therefore, this study suggests conducting long-term follow-up studies to regularly collect dynamic data on adolescents’ exercise types, executive function, and mobile phone dependence. By using statistical methods such as cross-lagged analysis, the direction of influence among the three can be clarified, and whether the improvement of executive function through open-skill exercises can continuously contribute to the reduction of mobile phone dependence over time can be verified, filling the gap that cross-sectional studies cannot validate the causal sequence.

Secondly, at the level of sample characteristics, the samples were only selected from freshmen and sophomores in three cities of Sichuan Province via convenience sampling, failing to cover different provinces and urban levels across the country, nor include adolescents of other age groups such as junior high school students and college students, making it difficult to represent the overall characteristics of the national adolescent group. Therefore, this study suggests breaking through the geographical limitations of Sichuan Province, including adolescents from different provinces and urban levels nationwide, and covering groups of different academic stages at the same time, so as to eliminate sample biases caused by a single regional education model and academic stage characteristics, and make the research results applicable to a broader adolescent group.

Thirdly, at the level of data collection, this study classified exercise types through student self-administered questionnaires, which are prone to recall bias and social desirability bias, thereby affecting the objectivity of exercise type classification. Meanwhile, this study did not collect data on students’ participation in non-sports activities such as music, dance, and foreign language learning. These activities may also affect executive function and mobile phone dependence levels, leading to the inability to rule out the interference of such factors and resulting in insufficient exclusivity of the research conclusions. In addition, this study only used the EFS-A to assess ecological executive function. Although it can reflect cognitive application in daily scenarios, it did not incorporate laboratory-standardized tasks. This prevents the study from capturing the fine-grained components of executive function, leading to an incomplete cognitive profile, and also makes it difficult to meaningfully compare the ecological executive function data of this study with the results of other studies that adopt laboratory measurements. Therefore, this study suggests supplementing laboratory-standardized tasks while retaining the EFS-A for assessing ecological executive function, so as to achieve a comprehensive evaluation of “daily application ability ++ basic cognitive mechanisms.” In addition, it is recommended to add surveys on adolescents’ participation in other types of activities (such as music, dance, and foreign language learning) to control the potential impacts of such activities on executive function.

## Conclusion

From the perspective of motor skill classification (open skills vs. closed skills), this study adopted a cross-sectional research design to explore the correlation between different types of exercises and adolescents’ executive function as well as mobile phone dependence. It was found that compared with adolescents participating in closed-skill exercises, those engaging in open-skill exercises showed better performance in inhibitory control and cognitive flexibility, and experienced lower physical and mental impacts caused by mobile phone dependence. In addition, inhibitory control and cognitive flexibility played a partial mediating role in the relationship between open-skill exercises and the physical and mental impacts of mobile phone dependence. This study provides theoretical support and practical guidance for adolescents in choosing appropriate exercise types, and also offers a scientific basis for formulating relevant policies to promote adolescents’ physical and mental health.

## Data Availability

The raw data supporting the conclusions of this article will be made available by the authors, without undue reservation.
